# Length of carotid stenosis predicts peri-procedural stroke or death and restenosis in patients randomized to endovascular treatment or endarterectomy

**DOI:** 10.1111/ijs.12084

**Published:** 2013-07-29

**Authors:** Leo H Bonati, Jörg Ederle, Joanna Dobson, Stefan Engelter, Roland L Featherstone, Peter A Gaines, Jonathan D Beard, Graham S Venables, Hugh S Markus, Andrew Clifton, Peter Sandercock, Martin M Brown, CAVATAS Investigators

**Affiliations:** 1 Stroke Research Group, UCL Institute of NeurologyLondon, UK; 2 Department of Neurology and Stroke Unit, University Hospital BaselBasel, Switzerland; 3 Department of Epidemiology and Population Health, Medical Statistics Unit, London School of Hygiene and Tropical MedicineLondon, UK; 4 Sheffield Vascular Institute, Northern General HospitalSheffield, UK; 5 Neurology Department, Royal Hallamshire HospitalSheffield, UK; 6 Centre for Clinical Neuroscience, St. George's University of LondonLondon, UK; 7 Department of Neuroradiology, St. George's HospitalLondon, UK; 8 Division of Clinical Neurosciences, Western General HospitalEdinburgh, UK

**Keywords:** carotid stenosis, endovascular treatment, endarterectomy, restenosis, atherosclerosis, plaque length

## Abstract

**Background:**

The anatomy of carotid stenosis may influence the outcome of endovascular treatment or carotid endarterectomy. Whether anatomy favors one treatment over the other in terms of safety or efficacy has not been investigated in randomized trials.

**Methods:**

In 414 patients with mostly symptomatic carotid stenosis randomized to endovascular treatment (angioplasty or stenting; *n* = 213) or carotid endarterectomy (*n* = 211) in the Carotid and Vertebral Artery Transluminal Angioplasty Study (CAVATAS), the degree and length of stenosis and plaque surface irregularity were assessed on baseline intraarterial angiography. Outcome measures were stroke or death occurring between randomization and 30 days after treatment, and ipsilateral stroke and restenosis ≥50% during follow-up.

**Results:**

Carotid stenosis longer than 0.65 times the common carotid artery diameter was associated with increased risk of peri-procedural stroke or death after both endovascular treatment [odds ratio 2.79 (1.17–6.65), *P* = 0.02] and carotid endarterectomy [2.43 (1.03–5.73), *P* = 0.04], and with increased long-term risk of restenosis in endovascular treatment [hazard ratio 1.68 (1.12–2.53), *P* = 0.01]. The excess in restenosis after endovascular treatment compared with carotid endarterectomy was significantly greater in patients with long stenosis than with short stenosis at baseline (interaction *P* = 0.003). Results remained significant after multivariate adjustment. No associations were found for degree of stenosis and plaque surface.

**Conclusions:**

Increasing stenosis length is an independent risk factor for peri-procedural stroke or death in endovascular treatment and carotid endarterectomy, without favoring one treatment over the other. However, the excess restenosis rate after endovascular treatment compared with carotid endarterectomy increases with longer stenosis at baseline. Stenosis length merits further investigation in carotid revascularisation trials.

## Introduction

Endovascular treatment (EVT) of carotid stenosis by percutaneous transluminal balloon angioplasty or insertion of a stent has emerged as an alternative to carotid endarterectomy (CEA). Randomized controlled trials have shown an increased risk of peri-procedural stroke associated with EVT compared with CEA, but the excess in stroke risk was largely restricted to patients above the age of 70 1,2. Besides demographic and clinical factors, anatomical features of carotid stenosis may also contribute to differences in treatment risk. In previous research, plaque ulceration was associated with peri-procedural complications in CEA, but not in EVT 3–6. The length of stenosis has been identified as a predictor of peri-procedural stroke in EVT 3,6,7. However, neither plaque irregularity nor length of stenosis has been included in subgroup analyses of randomized trials comparing EVT versus CEA, and their impact on long-term outcome remains unknown.

## Aims

We therefore analyzed all intraarterial carotid angiographies obtained at baseline in the Carotid and Vertebral Artery Transluminal Angioplasty Study (CAVATAS) to answer the following questions: (1) Do degree of carotid stenosis, length of stenosis, or irregular plaque surface predict procedural risks of EVT or CEA?; (2) Do these factors have an impact on the long-term durability of EVT or CEA in terms of recurrent ipsilateral stroke or carotid restenosis?; and (3) Is one treatment superior to the other in terms of safety or efficacy depending on anatomy of stenosis?

## Methods

### Patients, randomization, treatment, and follow-up

CAVATAS was a group of randomized, open, multicenter trials designed to evaluate risks and benefits of EVT in carotid and vertebral artery disease. In the main part of CAVATAS, 504 patients with predominantly symptomatic moderate or severe carotid stenosis who were suitable for either procedure were randomized in a 1:1 ratio to EVT or CEA at 22 academic and nonacademic centers in Europe, Australia, and Canada between March 1992 and July 1997. Randomization was done by computer at the Clinical Trial Service Unit in Oxford, UK, with a minimization algorithm which took account of center and time from last symptoms. Trial methodology and initial results, as well as outcomes up to 11 years after treatment, have been previously reported 8–10. In the initial phase of the study, EVT was performed by balloon angioplasty alone. Stents became available during the trial and were used in 26% of patients in the EVT group, at the discretion of collaborating interventionists. Approved cerebral protection devices were not available at the time of recruitment in CAVATAS. Collaborating surgeons used their preferred techniques for endarterectomy, either with primary closure of the arteriotomy or closure with surgical patches. Patients in CAVATAS were examined by an independent neurologist at baseline and followed up 1, 6, and 12 months after treatment and yearly thereafter. There was no predefined length of follow-up, but centers were encouraged to follow up patients for as long as the center and individual patients were willing to do so. Follow-up ended in November 2007, 10 years after the last patient had been recruited. For the present analysis, we included patients randomized to EVT or CEA with available digital subtraction angiography (DSA) at baseline. CAVATAS is a registered trial (ISRCTN01425573).

### Carotid imaging at baseline and during follow-up

Selective DSA in at least two views was specified as the carotid imaging method of choice before randomization, in order to establish the suitability of the lesion for either EVT or CEA. Alternatively, centers were allowed to randomize patients on the basis of consistent findings in noninvasive magnetic resonance or computed tomography-based angiography (MRA and CTA) and carotid duplex ultrasound (CDU). Due to limited image resolution of films obtained from MRA and CTA at the time of recruitment into CAVATAS, only patients examined with DSA were included in the present analysis.

CDU of the carotid arteries was carried out one-year after treatment as a minimum and at yearly intervals thereafter. In many centers, additional examinations were performed one- and six-months after treatment, if possible. If cerebrovascular events occurred during follow-up, additional CDU and angiography (MRA, CTA, or DSA) was performed at the discretion of the local investigators. As previously described, degree of stenosis on CDU was determined by one investigator (L. H. B.) who was blinded to treatment, based on standardized flow velocity criteria at the central study office 9, and expressed equivalent to NASCET angiography measures 11.

### Anatomical parameters of carotid stenosis

**Fig 1:**
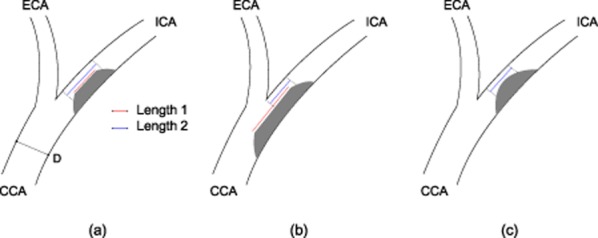
Measurement of length of stenosis. Stenosis length 1 (red line): distance between the two definite shoulders of the lesion. Stenosis length 2 (blue line): distance between the proximal and distal points where the degree of stenosis decreases to 80% of its maximum. Stenosis length is expressed as a fraction of the diameter (D) of the undiseased CCA. Examples a–c show length 1 and length 2 in different situations. (a) two definite lesion shoulders are present – length 1 and length 2 are similar; (b) two definite lesion shoulders are present but lesion proximally extends to carotid bifurcation – length 1 > length 2; (c) no definite lesions shoulders – only measurement of length 2 is possible. CCA, common carotid artery; ICA, internal carotid artery; ECA, external carotid artery.

A single, experienced investigator (L. H. B.) measured all DSA films obtained at baseline. The following parameters were assessed: *Degree of stenosis* was calculated according to the method used in NASCET 11. *Length of stenosis* was measured in two ways (Fig. 1): Length 1 was defined as the distance between the proximal and the distal shoulder of the stenotic plaque, in the projection that best elongated the stenosis 12. Length 2 was defined as the distance between the proximal and distal points where the degree of stenosis decreased to 80% of its maximum, regardless of whether definite lesion shoulders were present or not. To account for differences in scaling of DSA films, lengths 1 and 2 were expressed as a fraction of the diameter of the distal common carotid artery (CCA), which was measured. The distance between measurement of the CCA reference diameter and carotid bifurcation had to equate at least 2.5 times the CCA diameter, because previous research has shown that the diameter of the CCA stabilizes proximal to this point 13. We evaluated two definitions of length of stenosis: first, using length 1 whenever two definite lesion shoulders were present, and using length 2 if the plaque did not have two definite shoulders; and second, using always length 2. *Plaque surface* was classified as irregular or smooth 14. Irregular surface comprised ulceration (seen in profile as a crater extending from the lumen into a stenotic plaque, or on face view as a double density), or plaques with surface irregularity or presence of multiple small craters not classifying as ulcer niches 15.

### Outcome events

The safety end-point for the present analysis was death or any stroke causing neurological deficit lasting more than seven-days occurring between randomization and 30 days after treatment. The efficacy end-points were ipsilateral stroke lasting >seven-days occurring more than 30 days after treatment, and any residual or recurrent stenosis ≥50% or occlusion of the treated carotid artery on ultrasound during follow-up (termed restenosis). Outcome events were independently adjudicated in the central trial office by two investigators (J. E., LHB), blinded to the treatment. In case of disagreement, the principal investigator (M. M. B.) made the final adjudication.

### Statistical analysis

Patients were compared by the randomly allocated treatment (intention to treat). Receiver operator curves (ROC) were used to select the definition of length of stenosis (see above) which best predicted the safety end-point, and to define threshold values to dichotomize length and degree of stenosis for further analysis, at the point where sensitivity equalled specificity.

Within each treatment arm, the associations of degree and length of stenosis and plaque irregularity with the safety end-point were assessed on the univariate level with chi-square statistics; and on the multivariate level using binary logistic regression models. Cox regression was used to investigate the association between the three anatomical parameters and recurrent ipsilateral stroke. Impact on restenosis was tested using interval-censored generalized nonlinear models, as detailed previously 9. Formal testing for subgroup-treatment effect interactions was performed for each anatomical parameter and each end-point, by including a multiplicative interaction term in the binary logistic regression and the Cox regression models. Significant associations on the univariate level and interactions were adjusted for all anatomical parameters as well as age, sex, and baseline vascular risk factors (diabetes, hypertension, hypercholesterolemia, smoking, history of peripheral artery, and coronary heart disease). Effects on the safety end-point are expressed by odds ratios (OR), and effects on efficacy end-points are expressed by hazard ratios (HR). Provided in brackets are 95% confidence intervals of estimates.

### Role of the funding source

The study sponsors had no role in study design, data collection, data analysis, data interpretation, or the writing of the report. The corresponding author had full access to all the data in the study and had final responsibility for the decision to submit for publication.

## Results

**Fig 2:**
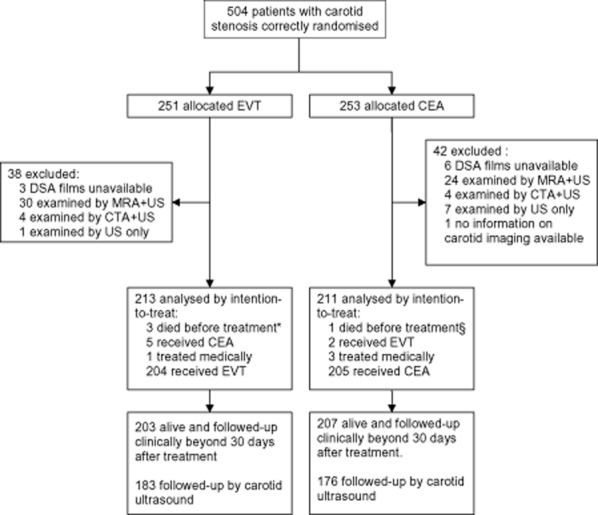
Study profile. *, 2 fatal strokes, 1 perforated duodenal ulcer. §, complications of preoperative cardiac pacing. EVT, endovascular treatment; CEA, carotid endarterectomy; DSA, Digital Subtraction Angiography; MRA, Magnetic Resonance Angiography; US, ultrasound; CTA, Computed Tomographic Angiography.

Imaging of carotid stenosis before randomization was done by DSA in 435 of all 504 patients randomized in CAVATAS (86%; EVT: 216/251, CEA: 217/253), MRA and CTA in 54 patients (11%) and 8 patients (2%), respectively, and ultrasound alone in 8 patients (2%). In one patient in the CEA arm, no information on the type of carotid imaging was available. In nine patients (EVT: *n* = 3, CEA: *n* = 6) examined by DSA, films were unavailable. The remaining 424 patients (EVT: *n* = 213, CEA: *n* = 211) with available DSA films were included in the analysis (Fig. 2). The two groups did not differ in age, sex distribution, proportion of patients with symptomatic stenosis (96%), presence of vascular risk factors, degree of stenosis, length of stenosis by either of the two definitions, or presence of an irregular plaque surface (Table 1). In the EVT group, 162 patients were treated with balloon angioplasty alone while stents were inserted in 51 patients.

**Table 1:** Baseline characteristics

	EVT (*n* = 213)	CEA (*n* = 211)
Age (years), *mean* ± SD	66.9 ± 8.2	66.9 ± 8.7
Male, *n* (%)	148 (70)	149 (71)
Ipsilateral cerebrovascular events within six-months before randomisation, *n (%)*	204 (96)	202 (96)
Vascular risk factors, *n* (%)		
Diabetes	31 (15)	29 (14)
Hypertension	111 (52)	115 (54)
Hypercholesterolemia	53 (25)	51 (24)
Smoking	162 (76)	160 (76)
Ischemic heart disease	85 (40)	84 (40)
Peripheral vascular disease	52 (24)	46 (22)
Degree of ipsilateral carotid stenosis,* mean ± SD	79.7 ± 13.0	77.8 ± 14.1
Length of ipsilateral carotid stenosis/CCA diameter		
Length 1, mean ± SD	0.76 ± 0.53	0.79 ± 0.53
Length 2, mean ± SD	0.66 ± 0.43	0.68 ± 0.42
Irregular plaque surface, *n (%)*	113 (53)	107 (51)

CCA, common carotid artery; CEA, carotid endarterectomy; EVT, endovascular treatment; SD, standard deviation. ^*^In percent, according to NASCET criteria.

**Fig 3:**
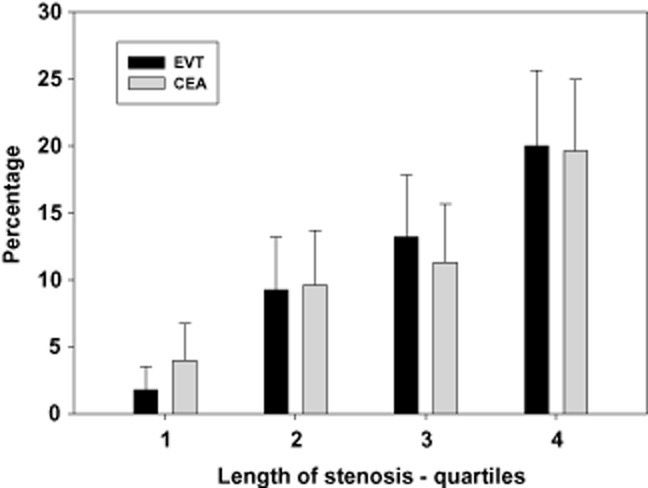
Peri-procedural stroke or death across quartiles of length of stenosis. Bars represent percentages of patients with the combined outcome event, vertical lines represent standard errors. See text for definition of length of stenosis. EVT, endovascular treatment; CEA, carotid endarterectomy.

There was no difference in the occurrence of death or stroke lasting >seven-days between randomization and 30 days after treatment between the EVT arm (23/213 patients, 10.8%: 13 nonfatal strokes, 9 fatal strokes, 1 nonstroke death) and the CEA arm (24/211 patients, 11.4%: 20 nonfatal strokes, 4 nonstroke deaths), OR 0.94 (0.52–1.72). In the entire study population, length of carotid stenosis using either definition significantly predicted the safety end-point [definition 1, i.e., using length 1 where possible and otherwise length 2: area under the ROC 0.62 (0.54–70), *P* = 0.008; definition 2, i.e., always using length 2: area under the ROC 0.67 (0.59–0.74), *P* < 0.001]. Definition 2 (always using length 2) was selected for further analysis. There was a continuous increase in risk of peri-procedural stroke or death across quartiles of increasing length of stenosis (by definition 2) in both arms, as shown in Fig. 3. The ideal threshold length for prediction of the safety end-point equalled 0.65 times the CCA diameter, with a sensitivity and specificity of 62%. For further analysis, length 2 was dichotomized at this value. Degree of carotid stenosis did not predict peri-procedural stroke or death (AUC 0.532, 95% CI 0.445–0.619, *P* = 0.469), and patients were therefore separated at the median value (81.25% stenosis) for further analysis.

**Fig 4:**
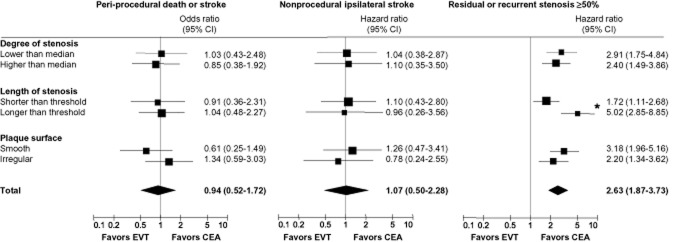
Comparison of peri-procedural stroke or death, nonprocedural ipsilateral stroke, and restenosis ≥50% between treatment arms, according to anatomical factors. Unadjusted odds ratios and hazard ratios with 95% confidence intervals (95% CI) of outcome events in EVT compared with CEA. Median degree of stenosis was 81% according to the method used in the NASCET trial. Threshold length of stenosis was 0.65 times the diameter of the distal common carotid artery, using the definition of length 2 (see text for details). *, *P* = 0.003 for interaction between length of stenosis, treatment, and restenosis. EVT, endovascular treatment; CEA, endarterectomy.

In patients with stenosis longer than the threshold length, the safety end-point occurred significantly more often than in patients with shorter stenosis, both in the EVT arm (17.1% vs. 6.9%, unadjusted OR 2.79 [1.17–6.65], *P* = 0.02) and in the CEA arm [16.5% versus 7.5%, unadjusted OR 2.43 (1.03–5.73), *P* = 0.04; Table 2]. Associations remained significant after adjustment for degree of stenosis, plaque irregularity, age, gender, and vascular risk factors [EVT: adjusted OR 3.10 (1.19–8.13), *P* = 0.02; CEA: adjusted OR 2.67 (1.07–6.68), *P* = 0.04]. There were no significant associations between degree of stenosis or surface irregularity with the safety end-point in both arms. There was no evidence that either treatment was safer than the other in the different anatomical subgroups (Fig. 4).

**Table 2:** Peri-procedural stroke or death, nonprocedural ipsilateral stroke, and restenosis ≥50%, stratified by degree of stenosis, length of stenosis, and plaque surface

	Peri-procedural stroke or death*			Nonprocedural ipsilateral stroke†			Ipsilateral residual or recurrent stenosis ≥50%†		
Degree of stenosis		OR (95% CI)	Degree of stenosis		HR (95% CI)	Degree of stenosis		HR (95% CI)
*n* (%)			*n* [5-year risk % (SE)]			*n* [5-year risk % (SE)]		
<Median	>Median		<Median	>Median		<Median	>Median	
EVT	10 (10.0)	13 (11.5)	1.17 (0.50–2.74)	7 [8.4 (3.3)]	7 [5.7 (2.5)]	0.92 (0.32–2.64)	46 [55.5 (5.8)]	53 [63.7 (6.1)]	1.12 (0.75–1.67)
CEA	11 (9.7)	13 (13.3)	1.42 (0.62–3.27)	8 [7.6 (2.8)]	5 [6.0 (2.6)]	0.78 (0.25–2.40)	23 [26.8 (5.1)]	26 [27.0 (6.3)]	1.41 (0.80–2.48)
	**Length of stenosis**			**Length of stenosis**			**Length of stenosis**		
**<Threshold**	**>Threshold**		**<Threshold**	**>Threshold**		**<Threshold**	**>Threshold**	
EVT	9 (6.9)	14 (17.1)	2.79 (1.17–6.65)‡ 3.10 (1.19–8.13)†§	10 [7.0 (2.6)]	4 [6.6 (3.2)]	0.78 (0.25–2.49)	55 [53.8 (5.8)]	44 [70.1 (6.1)]	1.68 (1.12–2.53)‡ 1.65 (1.08–2.51)‡§
CEA	9 (7.5)	15 (16.5)	2.43 (1.03–5.73)‡ 2.67 (1.07–6.68)‡§	8 [7.2 (2.7)]	5 [6.6 (2.9)]	0.84 (0.27–2.57)	32 [35.6 (5.5])	17 [23.4 (5.2)]	0.57 (0.32–1.04)
	**Plaque surface**			**Plaque surface**			**Plaque surface**		
**Smooth**	**Irregular**		**Smooth**	**Irregular**		**Smooth**	**Irregular**	
EVT	8 (8.0)	15 (13.3)	1.76 (0.73–4.25)	9 [9.1 (3.4)]	5 [4.7 (2.3)]	0.77 (0.44–1.34)	51 [61.3 (6.1)]	48 [39.3 (6.0)]	0.85 (0.57–1.27)
CEA	13 (12.5)	11 (10.3)	0.80 (0.35–1.85)	7 [8.1 (3.0)]	6 [5.8 (2.6)]	0.93 (0.54–1.60)	25 [29.7 (5.1)]	24 (35.1 (6.2)]	1.26 (0.72–2.22)

* Numbers in cells represent numbers of patients with end-point (percentage), and odds ratios (OR) with 95% confidence intervals (CI).

† Numbers in cells represent numbers of patients with end-point [cumulative percentage incidence five-years after treatment (standard error)], and hazard ratios (HR) with 95% CI. Median degree of stenosis was 81% according to the method used in the NASCET trial. Threshold length of stenosis was 0.65 times the diameter of the distal common carotid artery, using the definition of length 2 (see text for details).

‡ *P* < 0.05.

§ Adjusted for all anatomical parameters, age, gender, and vascular risk factors. Other OR and HR are unadjusted. EVT, endovascular treatment; CEA, carotid endarterectomy.

The 203 patients in the EVT arm and 207 patients in the CEA arm who were still alive 30 days after treatment were followed up clinically for a median duration of 5.0 years [interquartile range (IQR): EVT 3.0–6.6, CEA 2.9–6.0]. Nonperioperative ipsilateral strokes occurred in equal proportions of patients in both treatment arms [HR 1.07, (0.50–2.28)] with cumulative 5-year incidences of 6.9% each [standard error (SE): EVT 2.1% and CEA 2.0%]. Neither degree nor length of stenosis nor plaque irregularity was associated with nonperioperative ipsilateral stroke during follow-up in either treatment arm (Table 2), and there were no significant subgroup-treatment effect interactions (Fig. 4).

**Fig 5:**
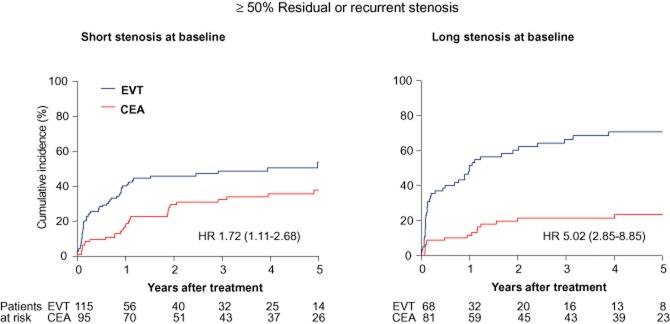
Cumulative incidence of restenosis ≥50% in both treatment arms, according to length of stenosis at baseline. Threshold length of stenosis was 0.65 times the diameter of the distal common carotid artery, using the definition of length 2 (see text for details). The interaction between length of stenosis, treatment, and restenosis was significant (*P* = 0.003). EVT, endovascular treatment; CEA, endarterectomy; HR, hazard ratio (95% confidence interval).

Ultrasound follow-up was performed in 183 patients in the EVT arm and 176 patients in the CEA arm, for a median duration of 3.9 (IQR 1.8–5.3) and 4.3 (IQR 1.4–5.6) years, respectively. Restenosis ≥50% occurred significantly more often among patients treated endovascularly (99 patients) than those having undergone CEA (49 patients), with cumulative 5-year incidences of 60.1% (SE 4.3%) and 31.4% (SE 4.0%), respectively, and an overall HR of 2.63 (1.87–3.73; *P* < 0.001). In the EVT arm, patients with long baseline stenosis had a higher risk of restenosis than those with short stenosis [cumulative 5-year incidences 70.1% versus 53.8%, unadjusted HR 1.68 (1.12–2.53), *P* = 0.01; adjusted HR 1.65 (1.08–2.51), *P* = 0.02, Table 2]. In contrast, baseline length of stenosis was not associated with restenosis in the CEA arm. There was a significant interaction between length of stenosis, treatment, and restenosis (adjusted and unadjusted *P* = 0.003), showing that the excess in restenosis after EVT compared with CEA was significantly greater among patients with long carotid stenosis than with short carotid stenosis at baseline (Figs 4 and 5).

As the derivation of the cut-off value for length of stenosis was based on outcome (peri-procedural stroke or death), we performed a sensitivity analysis dichotomizing length of stenosis (by definition 2) at the median value of the entire study population, which corresponded to 0.56 times the CCA diameter. Longer stenosis remained significantly associated with an increased risk of peri-procedural stroke or death in the EVT arm [short stenosis 6/110 patients, long stenosis 17/103 patients, OR 3.43 (1.29–9.07), *P* = 0.01], and by trend in the CEA arm [short stenosis 7/102 patients, long stenosis 17/109 patients, OR 2.51 (0.99–6.33), *P* = 0.05]. Also, the interaction between length of stenosis and treatment effect on restenosis remained significant.

## Discussion

The analysis of anatomical parameters of carotid stenosis and risk of treatment in CAVATAS yielded the following key findings: first, greater length of stenosis increased the risk for peri-procedural stroke or death in both EVT and CEA, to a similar degree; second, greater length of stenosis increased the risk for restenosis after EVT but was not associated with an increase in recurrent ipsilateral stroke; and 3 the degree of stenosis and plaque surface did not significantly alter treatment safety or efficacy of EVT or CEA.

CAVATAS was the first large-scale randomized trial comparing safety and efficacy of EVT versus CEA for patients with predominantly symptomatic carotid stenosis. The study protocol defined DSA as the method of choice for baseline carotid imaging, allowing for a detailed analysis of anatomical aspects of carotid stenosis prior to randomization. In more recent trials, only patients undergoing endovascular treatment had DSA examination of carotid stenosis as part of the stent procedure. Furthermore, follow-up was performed for up to 11 years after treatment and included regular examination by carotid ultrasound for the first time in a large trial of symptomatic carotid stenosis. This enabled us to investigate the relationship between anatomical parameters of carotid stenosis and long-term durability of EVT and CEA in terms of prevention of recurrent stroke and restenosis.

The length of stenosis was first identified as a risk factor in coronary artery stenting 12. Subsequently, two open cohort studies identified length of stenosis as a predictor of stroke in carotid artery stenting 3,7. In an analysis of preprocedural DSA in the stenting arm of the Endarterectomy Versus Angioplasty in Patients with Symptomatic Severe Carotid Stenosis trial, the association between length of stenosis, dichotomized at 10 mm, and risk of peri-procedural death or stroke was not significant 6. Length of stenosis in these studies was defined as the distance between the proximal and the distal shoulder of the atheromatous lesion. In the present study, we propose a new definition for measuring length of stenosis which is independent from the presence of definite lesion shoulders and which might be better suited to assess lesion length in the carotid bifurcation. In addition, we did an ROC analysis to determine the ideal threshold length of stenosis to predict complications. Our findings confirmed length of stenosis as a strong and independent risk factor for peri-procedural stroke or death in EVT. Patients with long stenosis may be at increased risk of embolism during EVT because of an increased likelihood of dislodging atherosclerotic debris or thrombus with larger plaque surface.

To date, research on anatomical risk factors in endarterectomy has focused on degree of carotid stenosis and plaque irregularity but did not investigate the significance of length of stenosis. The present study demonstrated for the first time that increasing length of stenosis leads to a very similar increase in treatment risk in CEA as in EVT. The mechanism for this increase in surgical risk remains unclear; it is conceivable that the larger carotid incision required in patients with long stenosis leads to increased activation of the coagulation system and thus a higher risk for thrombo-embolic stroke. As the risk increase for peri-procedural stroke or death is very similar in both treatments, length of stenosis is unlikely to help decide between the endovascular and the surgical approach in a patient in whom invasive revascularization is deemed necessary. However, length of stenosis – unlike degree of stenosis – appears to be a strong determinant of treatment risk and may thus help decide between invasive and conservative treatment in patients in whom the benefit of revascularization is uncertain. This question needs to be investigated in trials comparing conservative treatment versus revascularization by stenting or surgery in patients with low-to-intermediate risk carotid stenosis, such as the 2nd European Carotid Surgery Trial (ECST-2).

We previously reported a higher long-term risk of restenosis after EVT compared with CEA in CAVATAS 9. In the present analysis, increased length of stenosis at baseline independently predicted residual or recurrent carotid stenosis after EVT. A possible explanation for this finding is that mechanisms leading to residual or recurrent stenosis in EVT, such as elastic recoil of the artery wall, wall hematoma following injury of the intima, and neointimal hyperplasia, may be more pronounced if a longer lesion is treated endovascularly. In contrast, length of stenosis did not predict restenosis in patients treated with CEA. Length of stenosis thus helps identify patients at increased risk of restenosis with EVT who may benefit from prolonged ultrasound follow-up. However, the results warrant confirmation in modern trials using primary carotid stenting, where rates of severe restenosis have been reported to be much lower than in CAVATAS 16,17.

Our findings that the degree of stenosis was not associated with procedural risk of EVT or CEA were in accordance with previous case series and clinical trials 3–7,18. A meta-analysis of CEA trials identified plaque irregularity as an independent risk factor for surgical complications 5, a finding which we could not confirm.

Our study has important limitations. First, the majority of patients in the EVT arm were treated with balloon angioplasty alone, without use of stents. Since the time of recruitment in CAVATAS, primary stenting has replaced balloon angioplasty as the endovascular treatment technique of choice, potentially limiting the external validity of our findings. The limited number of patients in whom stents were inserted in CAVATAS (*n* = 51 were included in the present analysis) did not allow for investigation of the influence of the assessed anatomical parameters in carotid artery stenting. However, the observed association of length of stenosis and risk of EVT was consistent with findings from open registries of primary stenting. Our results need confirmation in recent and ongoing trials of carotid revascularization including carotid stenting. Second, the study may have been underpowered to detect interactions of stenosis morphology with treatment regarding recurrent stroke. Third, a single rater reviewed all films, and we therefore cannot provide data on interrater reliability of the measurements. However, because determination of length of stenosis is based on objective measurement, we would expect the results to be reproducible. Nevertheless, in order to generalize our findings to current clinical practice, the method needs validation especially in noninvasive angiography, which has now largely replaced intraarterial angiography in the diagnostic workup of carotid stenosis.

In conclusion, our study demonstrated for the first time that increasing length of stenosis increased the procedural risk of both EVT and CEA, to a similar degree. Patients with longer stenosis were at higher risk of restenosis after EVT, but length of stenosis was not associated with recurrent ipsilateral stroke. The results of our study therefore do not favor one treatment as a better choice over the other in patients with long carotid stenosis. Further research is warranted to investigate whether improved anti-thrombotic medication or specific interventional or surgical techniques (e.g., protection devices in EVT, shunting in CEA) or medication regimes might be effective in reducing the higher procedural risk in patients with long carotid stenosis. To confirm our findings, and to examine whether the results can be reproduced with noninvasive magnetic resonance or computed tomography-based imaging techniques, the association between length of stenosis and procedural risk, recurrent stroke, and restenosis should be investigated in recent and ongoing trials of carotid revascularization.

## CAVATAS investigators

*Organizing Committee*: J. M. Bland, M. M. Brown (principal investigator), T. Buckenham, A. Clifton, P. A. G. Sandercock, R. S. Taylor. *Data Monitoring Committee*: R. Collins, G. Tognoni, C. P. Warlow (chairman). *Trial Statistician*: J. M. Bland. *Trial Office Staff*: St George's Hospital Medical and UCL Institute of Neurology, UK: T. Bleakley, D. Colquhoun, L. Coward, F. Crawley, P. Dobinson, R. Featherstone, S. Holder, H. Markus, D. J. H. McCabe, A. Pereira, J. Rogers, L. Silver. Clinical Trial Service Unit, Oxford, UK: J. Burrett, J. Crowther, M. Dobson, B. Hafner, J. Heineman, C. Hope, S. Knight, A. Naughten, A. Radley, S. Richards, D. Smith, S. Wenzel. *Clinical Audit Committee*: M. Harrison, J. Ferro. *Credentials Committee*: J. Beard, T. Buckenham, M. M. Brown, A. Clifton.

*CAVATAS Centres randomizing patients with carotid stenosis between surgery and endovascular treatment (number of patients enrolled at each centre) and individual investigators. Australia*: Austin and Repatriation Medical Centre, Heidelberg (18) (C. F. Bladin, G. A. Donnan, G. Fell, G. Fitt, J. Royle); Royal Melbourne Hospital (1) (S. Davis, R. Gerraty, P. Mitchell); Royal Perth Hospital (21) (M. A. Goodman, G. J. Hankey, M. S. Khangure, M. M. Lawrence-Brown, J. Linto, W. McAuliffe, F. J. Prendergast, K. Siennarine, E. G. Stewart-Wynne). *Canada*: Ottowa General Hospital (2) (S. Grahovac, W. Morrish, N. Pageau, C. E. Pringle, D. M. Richard). *Germany*: Heinrich Heine University, Duesseldorf (4) (J. Malms, L. Reiher, M. Siebler). *Italy*: Policlinico St Marco, Bergamo-Zingonia (13) (G. Belloni, M. Porta). *Spain*: Hospital Clinic I Provincial, Barcelona (4) (A. Chamorro, N. Vila, V. Riambau, F. Vazquez); Hospital Universitario Virgen del Rocio, Sevilla (16) (F. Boza, J. L. Garcia Rodríguez, A. Gil Peralta, A. González, J. R. González Marcos, A. Mayol Deya, J. Rauno). *Switzerland*: University Hospital, Basel (5) (E. C. Kirsch, Ph. A. Lyrer, J. A. Rem); Centre Hospitalier Universitaire Vaudois, Lausanne (1) (J. Bogousslavsky, A. Uske). *United Kingdom*: Royal Hallamshire and Northern General Hospitals, Sheffield (193) (J. D. Beard, T. J. Cleveland, C. Doyle, P. A. Gaines, A. Sivaguru, G. S. Venables); London, Atkinson Morley's and St. George's Hospitals (100) (M. M. Brown, T. Buckenham, A. Clifton, D. Colquhoun, F. Crawley, P. W. Leopold, T. Loosemore, D. J. H. McCabe, A. Pereira, J. Rogers, R. S. Taylor); Walton Centre, Liverpool (4) (T. P. Enevoldson, G. Gilling-Smith, P. Harris, T. Nixon); London, King's College (21) (P. Baskerville, T. Cox, S. Fraser, M. Jeffrey, H. Markus, J. Molloy); London, Royal London (1) (P. Butler, J. Dick, F. Frankel); Western General Hospital, Edinburgh (31) (A. Bradbury, D. Collie, J. A. Murie, C. V. Ruckley, P. A. G Sandercock, D. Schultz, R. J. Sellar, J. Wardlaw); Withington Hospital, Manchester (7) (R. J. Ashleigh, C. N. McCollum, P. O'Neill); Newcastle General Hospital (14) (A. Gholkar, A. D. Mendelow, T. J. Walls); University Hospital of Wales, Cardiff (6) (H. Angus-Leppan, S. Halpin, J. Hughes, I. Lane, M. Wiles, A. M. Wood); Gloucestershire Royal Hospital (19) (P. A. Birch, J. J. Earnshaw, G. N. Fuller, B. Heather, K. Poskitt, A. J. Tottle); Queens Medical Centre, Nottingham (18) (D. T. Hope, D. Jefferson, N. McConachie); Queen Elizabeth Neuroscience Centre, Birmingham (5) (M. Duddy, M. T. E. Heafield, R. K. Vohra).
